# Effect of turmeric and triphala in the treatment of oral submucous fibrosis among Indians

**DOI:** 10.6026/973206300200851

**Published:** 2024-08-31

**Authors:** Praveen Kumar Singh, Balkrishn Gaur, Shabana Banu, Prathibha M, Trushna Rahangadale, Radhika Gupta, Shilpa Jain

**Affiliations:** 1 Department of Dentistry, KM Medical College, Mathura, Uttar Pradesh, India; 2 Department of Oral Medicine and Radiology KD Dental College and Hospital, Mathura, Uttar Pradesh, India; 3 Department of Oral Medicine and Radiology, The Oxford Dental College, Bangalore, Karnataka, India; 4 Department of Periodontology, Subbaiah Institute of Dental Sciences, Shimoga, Karnataka, India; 5 Department of Oral Medicine and Radiology, Index Institute of Dental Sciences, Indore, MP, India; 6 Department of Prosthodontics Crown and Bridge, College of Dental Sciences and Hospital, Rau, Indore, MP, India

**Keywords:** Anti-oxidant property, burning sensation, mouth opening, oral sub mucous fibrosisturmeric, triphala

## Abstract

Oral Submucous Fibrosis (OSMF) is a potentially malignant precancerous lesion. Various treatment modalities are tried to cure it including herbal medicaments. Therefore, it is of interest to determine whether using 1 gramme of triphala and 1 gramme of turmeric combined in a glycerine solution would be clinically effective in treating oral submucous fibrosis (OSMF). The study comprised thirty-six participants in the 18-50 age range who had no systemic problems. The medications prescribed for the patients were to be applied three to four times a day. Before and after therapy, blood samples were taken in order to check for any systemic outcomes of the drugs. Both before and after therapy, there were recordings of the burning feeling and mouth opening. For 4 months, patients were checked on a monthly basis after that. A statistical analysis was performed for the variations in the mouth opening and the changes in the burning sensation on the visual analogue scale (VAS). There was a statistically significant improvement in the mouth opening and burning sensation. Turmeric and triphala provide a natural product combination that is both safe and effective for treating OSMF symptoms.

## Background:

Fibrous bands forming in the submucosal layers are a defining feature of the oral cavity disorder known as oral submucous fibrosis (OSMF). This results in various symptoms as well as increasing difficulties opening the mouth. The fibrosis may worsen to the point where the afflicted parts harden and turn white, making it difficult to open the mouth wide and having restricted tongue mobility. If this lesion left untreated, can progress to malignancy (oral cancer) [1]. There is no known cause for Oral Submucous Fibrosis (OSMF), a persistent, mild condition that affects the oral mucosa. It is marked by a large amount of collagen tissue deposition within the submucosa, with sporadic dissemination to the oesophagus and throat. The symptoms of the disorder include trismus, burning feeling, and loss of gustatory sensitivity, loss of tongue mobility, and rigidity and blanching of the oral mucosa. Indian individuals are affected by the bulk of these cases [2]. Its prevalence in India varies between 0.20 and 0.5%, with a higher occurrence in the country's southern parts [3]. The exact cause of OSMF is unknown, but it is thought to be multifactorial due to a number of triggering factors (e.g., chewing areca nuts, eating chillies, nutritional deficiencies, and so on) that promote the disease's progression by inducing a juxta-epithelial inflammatory reaction in the oral mucosa. Chewing areca nuts may enhance the synthesis of connective tissue growth factor (CTGF) and, as a result of inflammation, the local concentration of transforming growth factor beta (TGF-β) [4]. Often chewed with betel leaf and other ingredients, areca nut mixtures are called by different names depending on the location; in India, they are called "pan" or "paan". Constant chewing and contact with these materials can cause long-term oral tissue irritation and inflammation, which can eventually result in fibrous bands forming and a limited mouth opening. The inability to eat, speak, and execute other oral tasks can be severely hampered by this illness. A delayed diagnosis can result from the modest and easily missed early signs of OSMF, which include nonspecific vesicular stomatitis and a burning sensation in the oral cavity [1]. The histological features associated with OSMF include atrophy or hyperplasia of the overlaying epithelium, juxta-epithelial fibrosis, buildup of hyalinized collagen beneath the basement membrane with a progressive loss of vascularity, and persistent inflammatory cell infiltration in the lamina propria. Patients with OSMF are categorised using clinical, functional, histological, and physiological diagnosis [5].

There are currently no conclusive therapy guidelines that are indicated for the management of OSMF, despite recent advances in pharmacotherapeutics [6]. An increasing number of people are interested in investigating complementary and alternative therapies for OSMF, especially those including herbal formulations. Phytotherapy, another name for herbal medicine, is a centuries-old method that uses substances derived from plants to cure and prevent a wide range of illnesses. Due to their apparent natural nature, low risk for side effects, and potential therapeutic advantages, the use of herbal formulations in the treatment of OSMF has attracted interest [7]. Herbal formulations for OSMF usually include a variety of plant-derived substances that have the potential to be therapeutic due to their bioactive components. With the goals of reducing fibrosis, improving oral opening, preventing disease development, and relieving symptoms, these formulations are frequently applied topically or orally. Many herbal substances, such as aloe vera, triphala, licorice root extract, ginkgo biloba, green tea, trikatu, and turmeric, have been studied for their possible effectiveness in treating OSMF. Additionally, honey was tested to cure OSMF [2, 8].

In traditional Ayurvedic medicine, triphala is a concoction of three fruits: haritaki, bibhitaki, and amla. It has anti-inflammatory, immunomodulatory, and antioxidant properties. For OSMF, triphala has been researched for possible therapeutic advantages. It strengthens the antioxidant action, lessens fibrosis, and boosts oral mucosal health [9].One popular herbal product is turmeric, which contains three curcuminoids: bisdemethoxycurcumin, curcumin, and demethoxycurcumin. Numerous health benefits of turmeric include its anti-inflammatory, antioxidant, blood-circulation-boosting, and anti-mutagenic properties [2]. Curcumin is the most studied of the active compounds called curcuminoids found in turmeric (Curcuma longa Linn). The primary bioactive ingredient in turmeric (curcumin) has strong antioxidant properties and the ability to scavenge or neutralise several reactive oxygen species that are created by macrophages, protecting cells from oxidative damage [10]. Therefore, it is of interest to develop herbal remedies like turmeric and triphala worked clinically in treating oral submucous fibrosis (OSMF).

## Materials and Method:

This using prospective observation study was done in the Department of Oral Medicine. Before beginning the study, the institutional ethics committee's consent was obtained. Every patient gave their informed consent to participate in the research. Thirty-six patients with newly diagnosed OSMF were chosen, and they were to be monitored after receiving herbal medicine treatment consisting of a combination of triphala and turmeric. Thirty-six people participated in the study. We purchased the herbal remedies triphala and turmeric from the neighbourhood market. Patients of all ages, genders, occupations, socioeconomic classes, ethnicities, and OSMF phases participated. Given the distinctive clinical characteristics of OSMF, the illness was identified clinically. The study excluded those individuals with systemic illnesses and those receiving OSMF medication already. All patients had a full blood investigation to determine their eligibility, and only those whose results were within normal ranges were added to the research. All of the chosen patients had incision biopsies taken in order to rule out dysplasia or overt cancer, as these cases had to be disqualified from the study. Every patient received encouragement to change their lifestyle and advice on how to stop using alcohol and smoke regularly. Every patient had a habit of some kind, but the majority chewed tobacco and consumed gutka. Yoga, meditation, behavioural therapy, and relaxation therapy were among the lifestyle adjustments. Glycerine was combined with one gram each of triphala and turmeric powder to create a paste. The patients were told not to eat or drink anything for the following fifteen minutes after applying this paste to their oral mucosa four to five times a day. In order to verify compliance and determine if all of the powder was utilised, the patients were requested to bring the used packets back to their follow-up appointments. In order to evaluate the mouth opening, the pre-treatment burning sensation was quantified using the visual analogue scale (VAS) and the interincisal distance was measured in millimetres (mm). Every patient was instructed to schedule follow-up appointments every month for duration of 4 months. Every time, the inter-incisal distance and the burning sensation on the VAS scale were noted. Following treatment, no biopsy was performed; instead, only clinical improvements-such as mouth opening and burning sensations-were considered.

## Results:

Thirty-six patients finished the research. The patients ranged in age from 18 to 50. The patients' average age was 27.57 ± 11.76 years. 28 men and 8 women made up the patient group. The mouth opening and burning feeling were noted during the initial visit. Both mouth opening and the burning sensation showed statistically significant improvements by the conclusion of treatment. Before and after the therapy, the mean burning sensation was 7.11±1.54 and 2.24±1.36, respectively (t = 13.47; P < 0.001). Before and after the treatment, the mean mouth opening was 22.32±5.0 mm and 28.35±3.42 mm, respectively (t = 8.56; P <0.001) (Table 1). A paired "t" test was used to examine the gender-wise differences in burning sensation and mouth opening, and the results showed that these differences were statistically significant (Table 2). Before therapy, the mean burning sensation in males was 7.12±1.56 on the VAS scale; and after therapy, it was 2.18±1.35. At t = 12.56 and P < 0.001, the improvement was statistically significant. In a similar vein, the mean burning sensation in females was 7.11±1.54 on the VAS scale prior to treatment; this improved to a mean of 2.24±1.36 (Table 2). There was a statistically significant improvement (t = 7.51; P < 0.001).In males, the mean mouth opening was 22.31±5.1 mm prior to therapy; it increased to 28.35±3.32 mm following therapy. Significant progress was made (t = 8.55; P < 0.001). In the same way, the mean mouth opening in females was 22.32±5.0 mm prior to therapy, but it improved to 25.46±3.35 mm (Table 2). At t = 2.75 and P = 0.02, the improvement was statistically significant. Data demonstrated that gender did not seem to have an impact on how responsive the treatment was. Patients receiving the same medication did not experience any clinically noteworthy side effects.

## Discussion:

The potentially cancerous condition known as oral submucous fibrosis (OSMF) is characterised by a gradual fibrosis of the oral mucosa, which makes it difficult to speak and eat and causes mucosal stiffness. Chewing tobacco and areca nuts is a typical behaviour linked to OSMF [8].Four months after using herbal medicine to treat OSMF, we saw improvements in mouth opening and a reduction in burning sensation in the current study. The patients did not experience any treatment-related side effects that were clinically noteworthy. Although recommended, medicinal and surgical treatment techniques have not proven effective in alleviating the symptoms thus far. Thus, the follow up for a successful therapy approach is currently ongoing. Numerous synthetic medications, herbal extracts, and natural plant extracts have been developed and tested for the treatment of OSMF [11]. In order to manage OSMF, Mobeen *et al.* examined the efficacy of a unique herbal paste formulation that included turmeric, tulsi (holy basil), and honey. They came to the conclusion that managing OSMF could benefit from the use of innovative herbal paste formulations [1]. Srivastava *et al.* examined the clinical effectiveness of herbal remedies (1 gm tulsi and 1 gm turmeric combined in glycerine base) for the treatment of OSMF, and their results were similar to ours. They came to the conclusion that a safe and effective combination of natural ingredients was provided by turmeric and tulsi for the symptomatic treatment of OSMF [2]. The effectiveness of turmeric and tulsi in treating oral submucous fibrosis was evaluated by Virani *et al.* They came to the conclusion that a combination of turmeric and tulsi could be a more effective treatment option than current methods for all grades of OSMF [12]. Similar to our study, Aich et al evaluated the efficacy of a new herbal mouthwash that contained triphala, honey, and curcumin in treating OSMF. They came to the conclusion that this trial has demonstrated the safety and effectiveness of a mouthwash containing curcumin, triphala, and honey in reducing OSMF symptoms [11]. Ingle's holistic research came to the conclusion that turmeric's strong anti-inflammatory and anti-oxidant pharmacological activities offer promise in the treatment of OSMF [13]. It has been demonstrated that Triphala Kwatha combined with Madhu is a more affordable, effective, safe, and superior medication for the treatment of Mukhapaka [14].

The systemic evaluation conducted by Gopinath *et al.* came to the conclusion that a number of medications were more effective than a placebo at improving mouth opening and burning sensation in patients with OSMF [6]. The usefulness of oil pulling in the treatment of oral submucous fibrosis (OSMF) was assessed by Shrivastava *et al.* They came to the conclusion that oil pulling can significantly reduce symptoms such as tongue protrusion and mouth opening [15]. Tulasi can be utilised as the primary therapy method in the care of patients with OSMF, according to the findings of a study conducted by Madhulatha *et al.* [16]. Turmeric has been used successfully to treat OSMF in a number of trials; however, in the current study, triphala and turmeric are combined to maximise the synergistic effects of both herbs. The current study has some limitations, including a smaller sample size, less scientific support, and inconsistent formulations. The body of research and our current understanding of herbal formulations in the treatment of OSMF serve as the foundation for suggestions in the future.

## Conclusion:

Turmeric and triphala provide a natural product combination that is both safe and effective for treating OSMF symptoms.

## Financial support and sponsorship:

Nil.

## Figures and Tables

**Table 1:** Improvement in the burning feeling and mouth opening

**Variables**	**Before the treatment**	**After the treatment**	**Paired 't' test**	***P value***
Mean mouth opening (mm)	22.32±5.0	28.35±3.42	8.56	<0.001
Mean burning sensation (VAS)	7.11±1.54	2.24±1.36	13.47	<0.001
VAS: Visual analogue scale

**Table 2:** Variations in mouth opening and burning sensation according to gender

**Gender**	**Variable**	**Before the treatment**	**After the treatment**	**Paired 't' test**	***P value***
Male	Mean mouth opening (mm)	22.31±5.1	28.35±3.32	8.55	<0.001
	Mean burning sensation (VAS)	7.12±1.56	2.18±1.35	12.56	<0.001
Female	Mean mouth opening (mm)	22.32±5.0	25.46±3.35	2.75	0.02
	Mean burning sensation (VAS)	7.11±1.54	2.24±1.36	7.51	<0.001
VAS: Visual analogue scale
